# Routine hemodialysis induces a decline in plasma magnesium concentration in most patients: a prospective observational cohort study

**DOI:** 10.1038/s41598-018-28629-x

**Published:** 2018-07-06

**Authors:** Nicoline H. J. Leenders, Frans J. van Ittersum, Tiny Hoekstra, Joost G. J. Hoenderop, Marc G. Vervloet

**Affiliations:** 1 Department of Nephrology, VU University Medical Center, Amsterdam, The Netherlands; 2 Department of Physiology, Radboud University Medical Center, Nijmegen, The Netherlands

## Abstract

In hemodialysis patients, lower plasma magnesium (Mg) concentrations are associated with a higher overall and cardiovascular mortality. The optimal concentration appears to be above the reference range for the healthy population. Plasma Mg is not routinely measured after hemodialysis. Aim of this study was to determine the effect of routine hemodialysis on plasma Mg. Plasma Mg was measured in duplicate before (Mg_pre_) and after (Mg_post_) dialysis in 6 consecutive hemodialysis sessions of 34 patients using a fixed 0.50 mmol/L dialysate Mg concentration. Mean Mg_pre_ was 0.88 mmol/L (±0.14) and mean Mg_post_ was statistically significantly lower: mean intra-dialytic decline 0.10 mmol/L (95%-CI 0.06–0.13). A 0.10 mmol/L higher Mg_pre_ was associated with a 0.03 mmol/L higher Mg_post_ (95%-CI 0.024–0.037). At a Mg_pre_ of 0.74 mmol/L, Mg_post_ equalled Mg_pre_. There was an intra-dialytic decline of plasma Mg at higher Mg_pre_ values and an increase at lower Mg_pre_ values. In conclusion, in the majority of the hemodialysis patients, Mg_pre_ concentrations are in the reference range of the healthy population, which may be too low for hemodialysis patients. Routine hemodialysis with the widely used 0.50 mmol/L dialysate Mg concentration, further declines magnesium in the majority of patients. Current dialysate Mg concentrations may be too low.

## Introduction

Magnesium is the fourth most abundant cation in the human body and is involved in many important physiological functions, including over 300 mainly ATP-generating enzymatic reactions, maintenance of secondary DNA and RNA structures, modulation of cell proliferation and regulation of transmembrane transport of other ions including potassium and calcium^1^. Magnesium homeostasis is dependent on intestinal absorption, uptake and release by bone, and renal excretion. In advanced stages of chronic kidney disease (CKD), plasma magnesium concentrations usually slightly increase as a consequence of reduced glomerular filtration^2^. In dialysis patients, clearance of magnesium becomes dependent on dialysis. For this reason, for a long time the general policy in dialysis patients has been avoidance of magnesium loading. However, serum magnesium concentration has been shown to be inversely associated with overall and cardiovascular mortality, incident coronary heart disease, incident atrial fibrillation and incident heart failure; and magnesium intake has been inversely associated with ischemic stroke in observational studies in the general population^3–8^. Magnesium inhibits vascular calcification in *in vitro* studies in vascular smooth muscle cells and in animal models of CKD^9,10^. In the last years, several observational cohort studies demonstrated that lower pre-dialysis plasma magnesium concentrations are independently associated with higher all-cause and cardiovascular mortality in hemodialysis patients^11–17^. In one Japanese observational study, the optimal concentration of magnesium was 1.27 mmol/L, a value well above the reference range for the healthy population (typically 0.70–1.00 mmol/L)^12^. This may point to a protective effect of slightly increased magnesium concentrations in hemodialysis patients.

In hemodialysis treatment, mostly, a fixed dialysate magnesium concentration is used and a dialysate concentration of 0.50 mmol/L is widely applied. However, there is no reliable evidence for this practice. Moreover, information from the literature on post-dialysis plasma magnesium concentrations and within-subject variability in patients on hemodialysis with a standard concentration of magnesium in the dialysate is insufficient. Few studies describe post-dialysis magnesium concentrations with a dialysate magnesium concentration of 0.50 mmol/L and generally an intra-dialytic decline was induced with post-dialysis magnesium concentration ranging between 0.67 to 1.09 mmol/L^18–21^. Those studies either had a very small sample size or measured magnesium concentrations at one dialysis sessions only. None of them described within-subject variability. Aim of this study was to determine the effect of modern routine hemodialysis treatment on post-dialysis plasma magnesium concentrations in chronic hemodialysis patients in 6 consecutive hemodialysis sessions. Secondary aim was to estimate the influence of pre-dialysis magnesium concentrations on this dialysis effect.

## Methods

### Location and population

This study was performed in Diapriva Dialysis Center, Amsterdam, The Netherlands, a university-affiliated dialysis clinic for chronic hemodialysis. The study was conducted in accordance with the declaration of Helsinki as revised in 2013 and was approved by the Ethical Committee of the VU University Medical Center (registration number 2016.275, NL57914.029.16). Possible candidates for study participation were adult patients on chronic intermittent hemodialysis or hemodiafiltration with a fixed dialysate magnesium concentration of 0.50 mmol/L and a stable regular 3-times weekly schedule since at least 3 months who provided written informed consent. Patients were excluded from participation if cessation of dialysis was expected within two weeks or if they received continuous intravenous magnesium supplementation with changed dose in the last two weeks or intermittent intravenous magnesium supplementation.

### Study design and data collection

A prospective observational cohort study was performed. The following baseline characteristics were collected: sex; age; length; weight; type of dialysis (hemodialysis or hemodiafiltration); usage, dose and type of oral magnesium supplements including laxatives and antacids, magnesium containing phosphate binders, proton pump inhibitors, diuretics, calcineurin inhibitors and intravenous magnesium supplementation; most recent estimation of dialysis efficiency (single pool Kt/V_urea_ per session according to Daugirdas’ formula); and most recent results of routine laboratory pre-dialysis (non-fasted) measurements for serum albumin, total calcium (albumin-corrected according to Payne’s formula), phosphate, intact parathyroid hormone (Chemiluminescant Microparticle Immuno Assay (CMIA), Architect system, Abbott diagnostics), hemoglobin and bicarbonate and pre- and post-dialysis measurements of potassium^22,23^.

Blood samples were taken before and immediately after hemodialysis at 6 consecutive hemodialysis sessions in 3 mL lithium-heparin gel tubes (BD-vacutainer). Processing of blood samples was performed, taking into account documented stability of magnesium concentrations in lithium-heparin plasma for at least 7 days if stored at 4–8 degrees and for 1 year if stored at −20 degrees Celsius^24^. Within one hour after collection, blood samples were centrifuged at 2000 × g during 15 minutes and plasma was temporarily stored at 4 degrees Celsius. All cryo vials were stored at −80 degrees Celsius within 0–3 days after collection until analysis. After collection of all samples, magnesium values were measured in duplicate with an automated analyzer from Roche-Hitachi using the colorimetric assay of the Cobas analysis kit. Assay variability for this assay has a coefficient of variation of 1.2% for within run precision (reproducibility) and 1.4% for between run precision (intermediate precision).

For every dialysis session, the following data were recorded: duration of dialysis, ultrafiltration volume, blood flow, dialysate flow, vascular access type and medication change in the mentioned categories.

### Outcome

Primary outcome was post-dialysis plasma magnesium concentration.

### Statistical analysis

Data were analyzed using SPSS for Windows (version 22.0; IBM SPSS Statistics. IBM Corp., Armonk, NY, USA). Continuous variables are described as mean and standard deviation for normally distributed variables and as median and interquartile range for non-normally distributed variables unless otherwise stated. Categorical variables are described as number and percentage. All available dialysis sessions of the study were used in the analysis, including sessions from participants of whom not 6 consecutive dialysis sessions were studied.

#### Assay variability and pre-dialytic intra-individual variability

Calculation of analytical variability and within-person total and biological variability was based on the methods described by Fraser and William that were recently updated by Braga *et al*.^25,26^. In short, analysis of variance techniques (ANOVA) were used to determine analytical variance and total pre-dialysis within-person variance. Subsequently, biological pre-dialysis within-person variance was calculated from these values using the formula described by Braga *et al*. The coefficient of variation (CV) was calculated by dividing the square roots of the variances by the corresponding means. We did not exclude outliers in order not to underestimate variance of results in clinical practice.

#### Magnesium concentration pre-dialysis and post-dialysis

For further analysis, the means of duplicate measurements were used.

To analyze the distribution of pre-dialysis and post-dialysis magnesium concentrations in the study population, for each patient the magnesium concentrations of the six dialysis sessions were pooled and the mean of these values was used. Pre-dialysis and post-dialysis magnesium are described as mean and standard deviation.

Statistical significance of the difference between pre-dialysis and post-dialysis magnesium was tested using the magnesium concentrations of each dialysis session in a linear mixed model for intra-dialytic change of magnesium. This model included only a fixed and random intercept using a scaled identity covariance matrix to account for random effects in the subjects. The estimate of the fixed intercept represented the mean intra-dialytic change.

#### Associations of pre- and post-dialysis concentrations

Linear mixed models were also used for the analysis of the association of post-dialysis magnesium concentration with pre-dialysis magnesium concentration. Another analysis was performed for the association of the intra-dialytic change of magnesium concentration with pre-dialysis magnesium concentration. In both analyses, the means of duplicate measurements of each dialysis sessions were used. Either a random intercept or a random slope or a random intercept plus a random slope was used, based on the lowest Akaike’s information criterion. A scaled identity covariance matrix was used to account for random effects in the subjects. The results were visualized in scatter plots showing the regression line resulting from the fixed effects in the linear mixed models. Second, other baseline characteristics and dialysis characteristics were added to the model for post-dialysis magnesium concentration to determine if this changed the association. Also, the association of post-dialysis magnesium with these factors was analyzed in individual models (not including pre-dialysis magnesium concentration).

#### Data availability

The datasets used during the current study are available from the corresponding author on reasonable request.

## Results

### Patients

**Figure 1:**
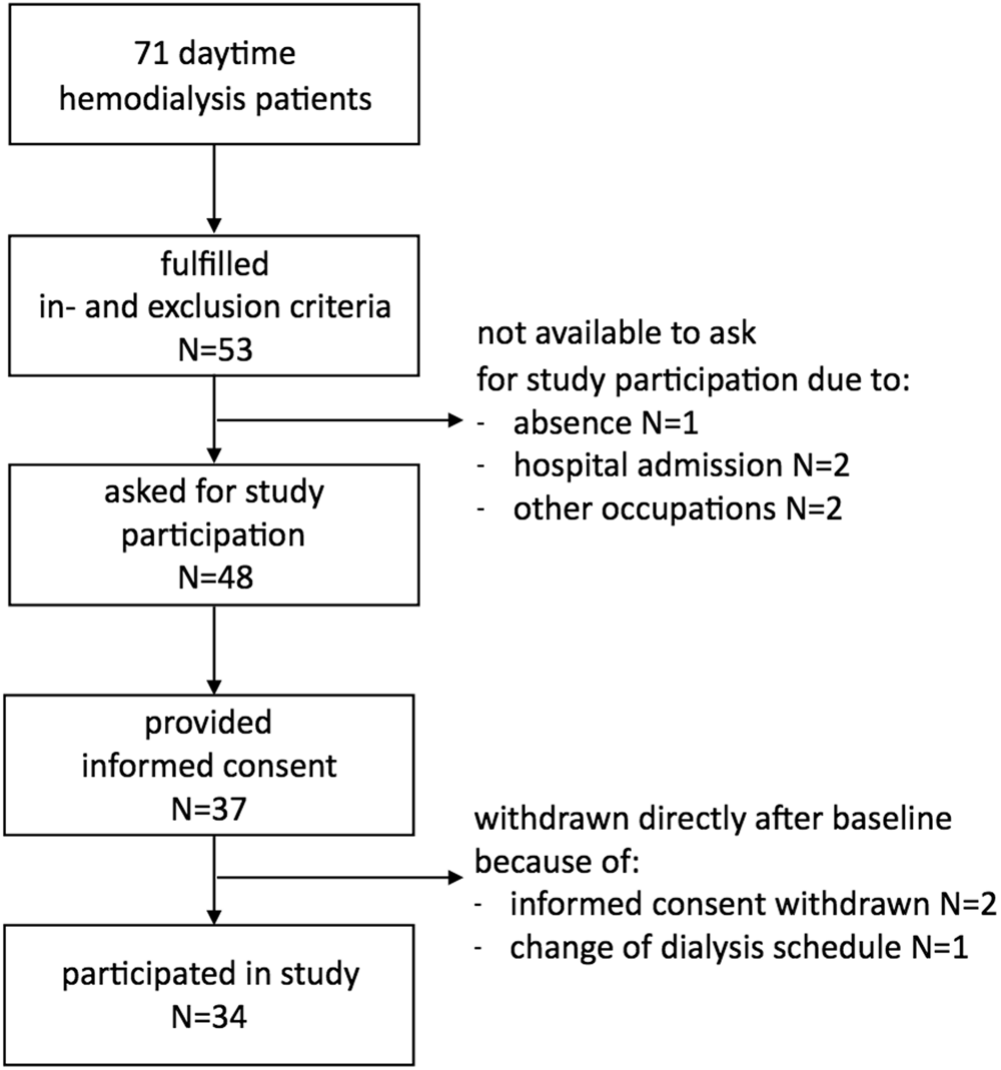
Flow-chart of patient enrolment in the study.

Details for patient enrolment are described in the flow-chart in Fig. 1. Finally, 34 patients participated in the study, collectively potentially encompassing 204 paired magnesium concentration values (six per patient).

In 7 patients, blood sampling was not performed after the first dialysis session of the study. One of those patients died during study follow-up before the 6^th^ dialysis session, due to a respiratory tract infection (not related to study procedures). In the other 6 patients, study follow-up was continued one extra (7^th^) dialysis session. In one other patient blood sampling was not performed after the second dialysis session. Two patients were studied only 2 dialysis sessions because thereafter their dialysis schedule changed. All other patients were studied 6 consecutive dialysis sessions.

### Baseline characteristics

Baseline characteristics are shown in Table 1. Participants had a median age of 68 years (Q1-Q3 58–75) and 68 percent were male. Most patients were treated with regular hemodialysis and seven patients (21%) were on on-line hemodiafiltration. Residual diuresis was present in twenty-one patients (62%) and median volume of diuresis in the total study population was 400 mL per day (Q1-Q3 0–1000). One patient had chronic diarrhea. In 5 patients (15%), a central venous catheter was used for vascular access. Mean dialysis efficiency (Kt/V_urea_) was 1.36. Proton pump inhibitors were used in 74% of patients, three patients (9%) used any medication containing magnesium, eighteen patients (53%) used diuretics and one patient used a calcineurin inhibitor. None of the patients received any type of intravenous magnesium supplementation. Mean dialysis duration during the study (not shown in Table 1) was 3.8 hours (95%-CI 3.6–3.9).

**Table 1:** Baseline characteristics of all 34 study patients.

Age (years)	68 (58–75)
Male	23 (68)
Body weight (kg)	73.6 (14.8)
Height (cm)	170 (11)
Residual diuresis (N (%))	21 (62)
Residual diuresis volume (mL)	400 (0–1000)
Chronic diarrhea	1 (3)
HDF	7 (21)
Vascular access type	
Fistula/Graft	29 (85)
Catheter	5 (15)
spKt/V (per session)	1.36 (0.40)
Laboratory parameters pre-dialysis	
PTH (pmol/L)	25.0 (8.7–42.0)
Calcium (mmol/L)	2.27 (0.15)
Albumin (g/L)	40.3 (3.3)
Phosphate (mmol/L)	1.64 (0.51)
Hemoglobin (mmol/L)	7.0 (0.7)
Bicarbonate (mmol/L)	22.6 (3.1)
Potassium (mmol/L)	5.2 (0.8)
Laboratory parameter post-dialysis	
Potassium (mmol/L)	3.7 (0.4)
Medication	
PPI	25 (74)
Oral Mg supplements/laxatives	1 (3)
Mg containing phosphatebinder	2 (6)
Intravenous Mg	0 (0)
Diuretic	18 (53)
CNI	1 (3)

Note: Categorical variables are expressed as number (percentage); continuous variables are expressed as mean (standard deviation) if normally distributed or median (Q1-Q3) if non-normally distributed. HDF, hemodiafiltration; spKt/V, single pool dialysis efficiency per session calculated with Daugirdas’ formula; PTH, parathyroid hormone measured with chemiluminescent microparticle immune assay from Abbott diagnostics, upper limit of reference range is 7 pmol/L; Calcium, total calcium adjusted for serum albumin; PPI, proton pump inhibitor; Mg, magnesium; CNI, calcineurin inhibitor.

### Variability

The analytical coefficient of variation calculated from all duplicate determinations of pre-dialysis and post-dialysis magnesium concentrations was 0.9%. The coefficient of variation was 5.7% for total intra-individual variability of pre-dialysis magnesium values. Based on these values, the coefficient of biological intra-individual variability of pre-dialysis plasma magnesium concentrations was 5.6%.

### Plasma magnesium concentrations

**Figure 2:**
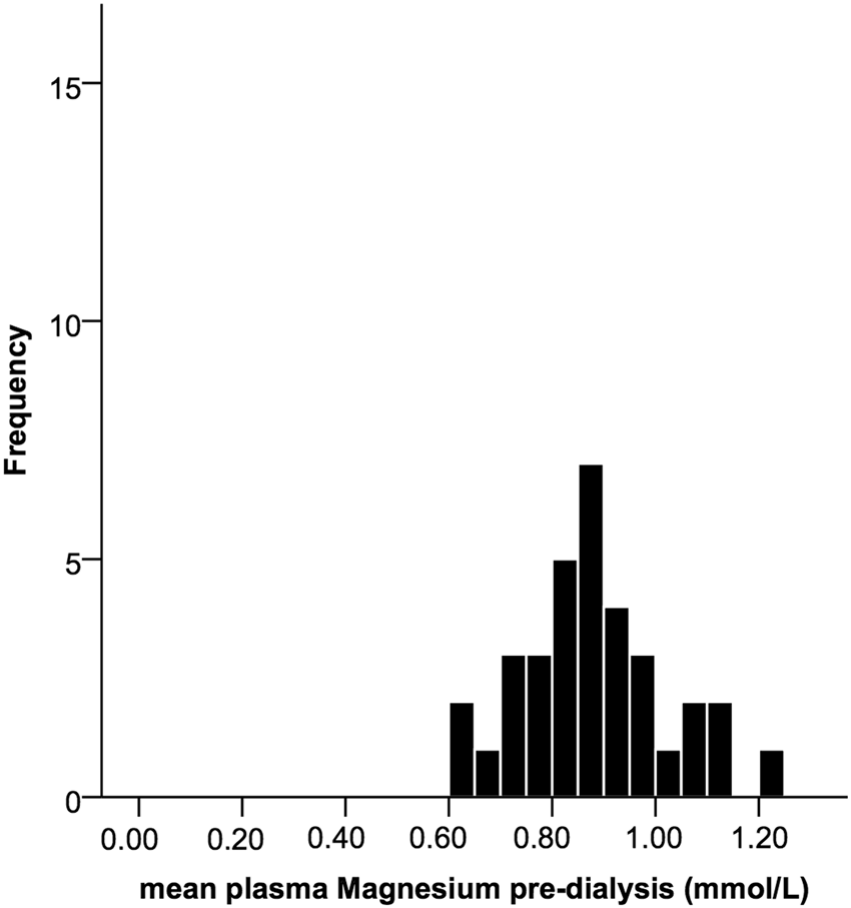
Distribution of pre-dialysis plasma magnesium concentrations in the individuals. The concentrations are the means of pooled values of the dialysis sessions in an individual.

**Figure 3:**
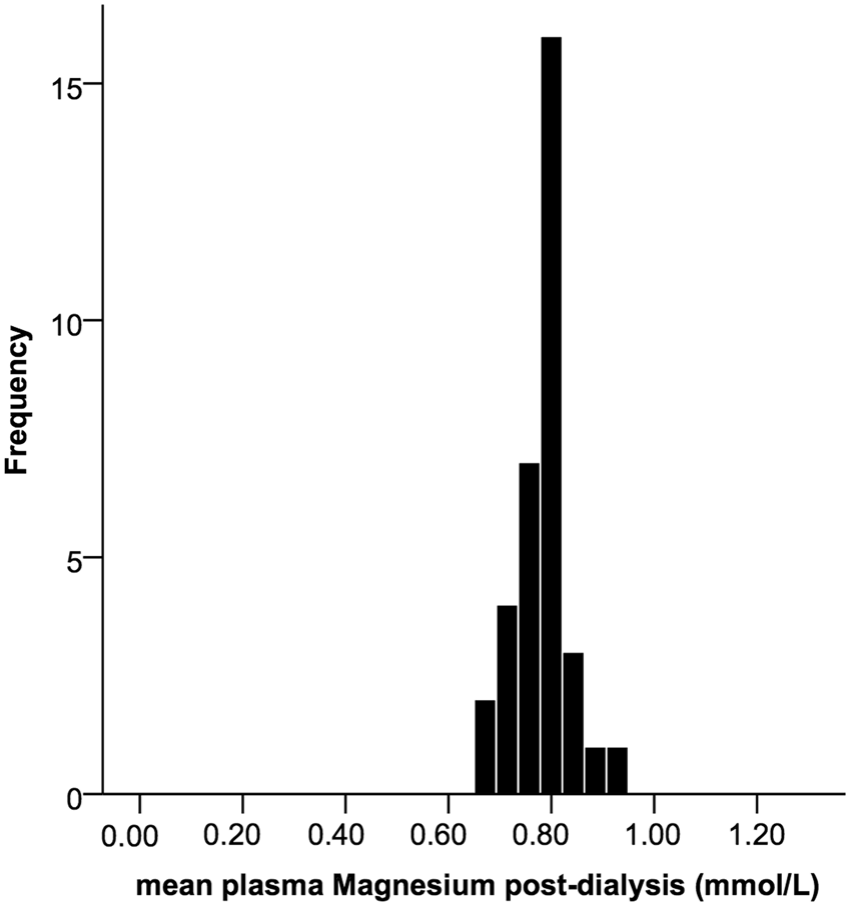
Distribution of post-dialysis plasma magnesium concentrations in the individuals. The concentrations are the means of pooled values of the dialysis sessions in an individual.

Mean pre-dialysis magnesium concentration was 0.88 (±0.14) mmol/L (Fig. 2). Mean post-dialysis concentration was lower with a smaller standard deviation: 0.78 (±0.05) mmol/L post-dialysis (Fig. 3). Magnesium concentrations changed significantly during dialysis with a mean decline of 0.10 mmol/L (95%-CI 0.06–0.13).

### Association between pre-dialysis and post-dialysis plasma magnesium

**Figure 4:**
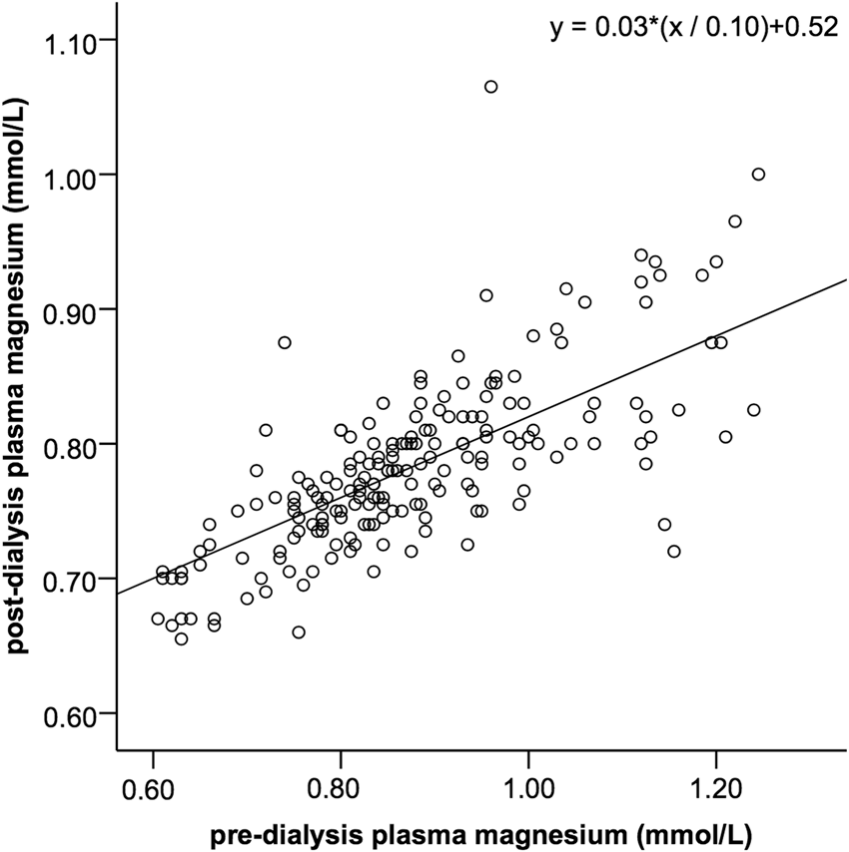
Association of plasma magnesium pre-dialysis with plasma magnesium post-dialysis. The regression line resulting from the linear mixed model is shown.

**Figure 5:**
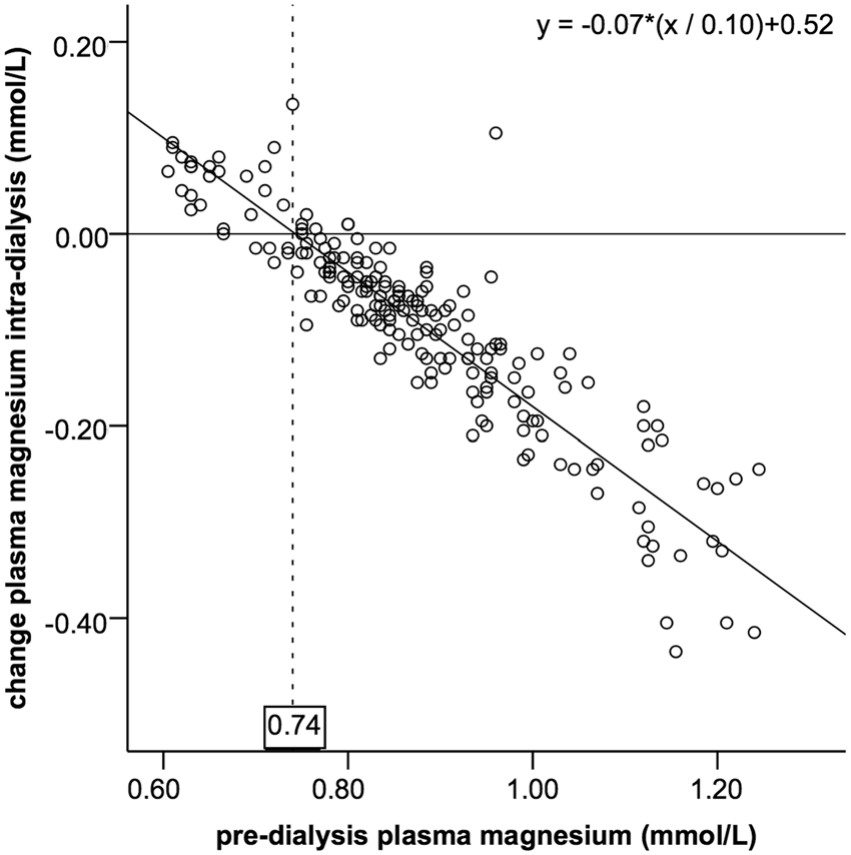
Association of plasma magnesium pre-dialysis with the intra-dialytic plasma magnesium change. The regression line resulting from the linear mixed model is shown. The regression line crosses the X-axis (no intra-dialytic change) at a pre-dialysis magnesium concentration of 0.74 mmol/L.

A 0.10 mmol/L higher pre-dialysis plasma magnesium was associated with a 0.03 mmol/L higher post-dialysis plasma magnesium (95%-CI 0.024–0.037) (Fig. 4). The post-dialysis magnesium concentration equalled the pre-dialysis magnesium concentration (no intra-dialytic change) at a pre-dialysis magnesium concentration of 0.74 mmol/L. There was an intra-dialytic decline of plasma magnesium at higher pre-dialysis plasma magnesium values and an increase of plasma magnesium at lower pre-dialysis values (Figs 4 and 5). The intra-dialytic change of magnesium was 0.07 mmol/L more negative for every 0.10 mmol/L increase of pre-dialysis plasma magnesium concentration (95%-CI 0.064–0.076) (Fig. 5). Baseline factors gender, age, serum albumin, hemoglobin, venous bicarbonate, height or body weight did not change the association of pre-dialysis magnesium concentration with post-dialysis magnesium concentration. In addition, the dialysis characteristics vascular access type, dialysis duration, ultrafiltration volume, blood flow, hemodiafiltration and dialysis efficiency did not modify this association. Of these factors, only ultrafiltration volume (UF) was statistically significantly associated with post-dialysis magnesium (post-dialysis magnesium was 0.02 higher (95%-CI 0.011–0.037) per 1,000 mL increase of UF).

## Discussion

Routine hemodialysis induces an intra-dialytic decline in plasma magnesium concentration in the majority of patients. This decline is higher if pre-dialysis magnesium concentration is higher. On average, there is a decline of plasma magnesium concentration during dialysis if pre-dialysis magnesium concentration is above 0.74 mmol/L. Correction for baseline factors and dialysis characteristics did not change the relation between pre-dialytic and post-dialytic magnesium concentration. In univariable analyses of these factors, only ultrafiltration volume had a statistically significant but weak association with post-dialytic magnesium concentration. This study demonstrates that pre-dialysis plasma magnesium concentrations in the majority of hemodialysis patients are in the reference range for the healthy population (typically 0.70–1.00 mmol/L) and in this study the mean concentration was 0.88 mmol/L (±0.14).

The plasma magnesium concentration in our study is lower compared to other studies that have reported post-dialysis plasma magnesium concentrations in hemodialysis with a dialysate magnesium concentration of 0.50 mmol/L^18–20^. Those studies showed variable results for mean post-dialysis plasma magnesium ranging from 0.84 to 1.09 mmol/L. Pre-dialysis plasma magnesium concentrations in these studies were also higher than in our study and ranged from 0.97 to 1.24 mmol/L. The different results may be explained by a change of magnesium balance in the past years due to increased use of proton pump inhibitors (in our study 74% of patients) and processed foods that have a low magnesium content^27–29^. Indeed, the highest concentrations were observed in the oldest study^18^. Moreover, reliability of previous studies was limited by either a small sample size or measurements at one dialysis session only.

We only included patients on hemodialysis with a fixed dialysate magnesium concentration of 0.50 mmol/L. Two previous studies that described pre- and post-dialysis magnesium concentrations when using a dialysate magnesium concentration of 0.75 mmol/L reported a stable mean plasma magnesium concentration during dialysis ranging 1.10–1.21 mmol/L^21,30^. One of these studies included only 14 patients and the other study measured concentrations in 46 patients but only at one dialysis session.

Strengths of this study are that the sample size was adequate, magnesium concentration was measured pre- and post-dialysis at multiple consecutive hemodialysis sessions with a fixed dialysate magnesium concentration of 0.50 mmol/L and also intra-individual variability was determined. Moreover, this study was performed in a center representing an average hemodialysis population in the modern Western hemodialysis practice and therefore takes into account modern dialysis techniques, Western diet and current medication use.

The study also has some limitations. First, total magnesium concentration was measured, not ionized magnesium, while especially the ionized (free) magnesium is amenable for dialytic clearance. However, observational studies on prognosis in hemodialysis patients measured total magnesium and showed that total magnesium concentration was relevant in this regard^11–16,27,31^. Importantly, it is more likely that the relative decline of ionized magnesium in this study is even more significant, which would strengthen and not weaken the main finding. A study that measured pre-dialysis ionized and total plasma magnesium concentration showed that in hemodialysis compared to healthy controls the ionized fraction of serum magnesium and the ionized magnesium concentration were decreased^32^.

Second, multiple baseline and dialysis characteristics did not confound the association of pre- and post-dialysis magnesium concentration in the analysis. However, in these patients on routine hemodialysis, for some of these factors, including dialysis duration and albumin, there was little variation amongst the study participants. Therefore, the results of the current study may have limited external validity for populations with higher variability of these potentially important factors.

Third, only patients with a fixed dialysate magnesium concentration of 0.50 mmol/L were included in this study.

In one study in a Japanese population with higher ranging magnesium levels, the relation between pre-dialysis magnesium concentration and mortality was J-shaped and the optimal concentration was 1.27 mmol/L^12^. In the current study all patients had concentrations below this concentrations, which also somewhat limits the external validity.

The results of this study may be important since a higher pre-dialysis plasma magnesium concentration is associated with a lower overall mortality, cardiovascular mortality and sudden death in multiple observational studies in hemodialysis patients and this association was present also at magnesium concentrations above the reference range of the healthy population^11,13–16,27,31^. In the current study, all patients had pre-dialysis magnesium concentrations below this optimum and routine hemodialysis declined the concentrations even more in most patients. In hemodialysis patients, especially without residual kidney function, removal of magnesium from the body is mainly dependent on dialysis. Thus, in patients with high pre-dialysis magnesium concentrations, a decline of plasma magnesium concentration during dialysis is the goal. However, our study results show that there is also a decline of magnesium during dialysis in patients with pre-dialysis magnesium concentration below the concentration that was optimal in the study by Sakaguchi *et al*. which was 1.27 mmol/L. Importantly the vast majority of our patients actually were below this value pre-dialysis. An additional decline of magnesium due to dialysis may be suboptimal. Information on the relevance of post-dialysis magnesium concentrations in relation to mortality in hemodialysis patients is lacking. However, if magnesium has direct protective effects it is conceivable that magnesium exposure over time is of importance instead of its pre-dialysis value only. Therefore, dialysis induced changes in its concentration may be of importance. Regarding the pre-dialysis concentration that may be suboptimal and the dialysis induced decline in most patients, current widely used dialysate magnesium concentration of 0.50 mmol/L may therefore be too low. This low dialysate magnesium concentration was implemented in dialysis practice in the past to prevent pre-dialysis hypermagnesemia in patients that are dependent on dialysis clearance. Maybe, a single study that reported less bone disease if hypermagnesemia was reduced in hemodialysis patients has also stimulated the use of low dialysate magnesium concentrations^18^. The observational prognostic data in hemodialysis cohorts that became available in recent years indicate that lower magnesium concentrations instead are associated with poor outcome. Moreover, in the modern European population increased use of processed foods and proton pump inhibitors reduce magnesium intake and bowel uptake and may have changed magnesium balance in hemodialysis patients resulting in a lower prevalence of pre-dialysis hypermagnesemia in hemodialysis patients^27–29^.

Currently, there are insufficient data to conclude what is the optimal plasma magnesium concentration, how this concentration must be regulated and how to guide patient tailored choices of dialysate magnesium concentration. In a single arm intervention study that increased dialysate magnesium concentration from 0.50 to 0.75 mmol/L, pre-dialysis ionized magnesium concentration increased statistically significant from 0.53 (±0.12) to 0.66 mmol/L (±0.02) after 24 months without clinical signs of hypermagnesemia and no toxic levels of magnesium were detected^33^. In a retrospective observational study in 45 hemodialysis patients in which dialysate magnesium concentration had been switched from 0.50 to 1.00 mmol/L, the frequency of intra-dialytic hypotension was lower in the 12 months after this switch compared to the 12 months before this switch^34^. Equally, in a small cross-over intervention study in 14 hemodialysis patients, intra-dialytic hypotension was less frequent with dialysate magnesium concentration 0.75 mmol/L compared to 0.50 mmol/L and 0.25 mmol/L^21^. Moreover, in a recent observational cohort study, adjusted all-cause and cardiovascular mortality rate were lower in a group with a dialysate magnesium concentration of 0.75 mmol/L compared to a matched group with a dialysate magnesium concentration of 0.50 mmol/L^35^. Of note, this study may have been biased by unknown center specific differences as all patients from the high dialysate magnesium group were recruited from one center and pre-dialysis ionized serum magnesium concentration was not significantly associated to all-cause mortality in univariate analysis. Future controlled intervention studies are needed to determine how plasma magnesium concentrations can be optimized by changing dialysate magnesium concentrations, and to find the optimal plasma magnesium concentration for morbidity and mortality.

In conclusion, pre-dialysis plasma magnesium concentrations may be too low in most patients on hemodialysis. Moreover, routine hemodialysis generally induces a further decline of plasma magnesium concentration. Current widely used dialysate magnesium concentration may be too low. Controlled intervention studies are needed to optimize magnesium balance in individual hemodialysis patients.
